# The Differential Distribution of RAPTA-T in Non-Invasive and Invasive Breast Cancer Cells Correlates with Its Anti-Invasive and Anti-Metastatic Effects

**DOI:** 10.3390/ijms18091869

**Published:** 2017-08-29

**Authors:** Ronald F. S. Lee, Stéphane Escrig, Catherine Maclachlan, Graham W. Knott, Anders Meibom, Gianni Sava, Paul J. Dyson

**Affiliations:** 1 Institute of Chemical Sciences and Engineering, Swiss Federal Institute of Technology (EPFL), CH-1015 Lausanne, Switzerland; ronald.lee@epfl.ch (R.F.S.L.); paul.dyson@epfl.ch (P.J.D.); 2 Laboratory for Biological Geochemistry, Ecole Polytechnique Fédérale de Lausanne (EPFL), CH-1015 Lausanne, Switzerland; stephane.escrig@epfl.ch (S.E.); anders.meibom@epfl.ch (A.M.); 3 Interdisciplinary Centre for Electron Microscopy, Ecole Polytechnique Fédérale de Lausanne (EPFL), CH-1015 Lausanne, Switzerland; catherine.maclaclan@epfl.ch (C.M.); graham.knott@epfl.ch (G.W.K.); 4 Center for Advanced Surface Analysis, Institute of Earth Sciences, University of Lausanne, CH-1015 Lausanne, Switzerland; 5 Callerio Foundation Onlus, via A. Fleming 22, 34127 Trieste, Italy

**Keywords:** breast cancer, invasion, metastasis, ruthenium

## Abstract

Nanoscale secondary ion mass spectrometry (NanoSIMS) combined with transmission electron microscopy (TEM) can be a powerful approach to visualize the exact distribution of drugs at the sub-cellular level. In this work, we exploit this approach to identify the distribution and localisation of the organometallic ruthenium(II)-arene drug Ru(η^6^-C_6_H_5_Me)(pta)Cl_2_, termed RAPTA-T, in MDA-MB-231 and MCF-7 human breast cancer cells. These cell lines have been chosen because the former cell lines are highly invasive and resistant to most chemotherapeutic agents and the latter ones are very sensitive to hormonal-based therapies. In the MDA-MB-231 cells, RAPTA-T was found to predominantly localise on the cell membrane and to a lesser extent in the nucleolus. These findings are consistent with the previously reported anti-metastatic properties of RAPTA-T and the observation that once internalized RAPTA-T is associated with chromatin. RAPTA-T shows a lack of membrane accumulation on the non-invasive MCF-7 cells, which correlates well with its selective anti-metastatic properties on invasive cell lines.

## 1. Introduction

**Scheme 1:**
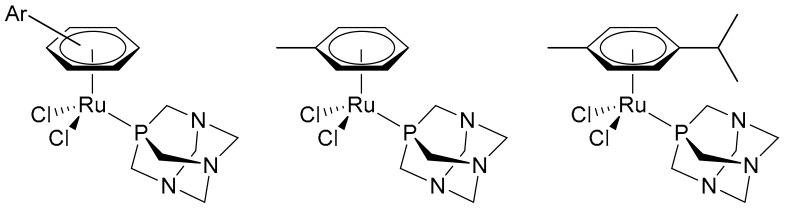
Generic RAPTA (Ru(η^6^-arene)(pta)Cl_2_) structure (left) and the structures of RAPTA-T (centre) and RAPTA-C (where the arene = *p*-cymene) (right).

Platinum-based drugs are widely used in the clinic [1,2]. However, in recent years, an increasing number of ruthenium complexes, with profoundly different properties compared with the currently used platinum drugs, e.g., higher cancer cell selectivity leading to reduced side-effects in vivo [3], have been (pre-)clinically evaluated [4,5,6,7]. All these drugs possess the classical coordination complexes structure, but there is now considerable interest in the anticancer properties of organometallic complexes, i.e., those containing direct metal-to-carbon bonds [8,9]. Of these organometallic compounds, the ruthenium(II)-arene drugs (Scheme 1), Ru(η^6^-arene)(pta)Cl_2_ where pta = 1,3,5-triaza-7-phosphaadamantane, termed RAPTA compounds, are the most advanced in pre-clinical studies, and many derivatives have been prepared and tested [10]. Specifically, Ru(η^6^-C_6_H_5_Me)(pta)Cl_2_ (RAPTA-T) possesses anti-metastatic properties in an in vitro model mimicking the detachment, invasion, migration, and re-attachment steps of metastasis formation [11]. This effect is much more evident on the invasive MDA-MB-231 breast cancer cells than on non-invasive MCF-7 breast cancer cells [11]. The in vitro studies were validated in an in vivo syngeneic, spontaneously metastasizing mammary carcinoma murine model, which showed RAPTA-T treatment to be effective, resulting in a reduction of lung metastasis formation of these tumours [12].

RAPTA-T is not the only compound of this family that has the capacity to reduce metastasis formation in experimental models [12]. However, RAPTA-T has other favourable physico-chemical and biological characteristics, i.e., particularly good water solubility and an intrinsic cancer cell selectivity demonstrated by a cytotoxicity difference between tumorigenic (74 μM) and non-tumorigenic (>1000 μM) cells [12], making it suitable for pharmacological development. Nevertheless, the development of RAPTA-T is also dependent on knowledge about its biological and pharmacological mode of action. Although RAPTA-T was not derived from a targeted approach, but essentially from the upgrading of clinically used platinum drugs [13], its mode of action is profoundly different, binding preferentially to proteins rather than DNA [14]. It is therefore necessary to acquire as much data as possible on the behaviour of RAPTA-T in cells as a function of cellular characteristics and of their response to treatment.

An approach that produces visual distribution maps of metal-based drugs in cells, nanoscale secondary ion mass spectrometry (NanoSIMS) [15] is attracting increasing attention [16] and has been used to image RAPTA-T in cisplatin-resistant human ovarian cancer (A2780CR) cells [17]. Consequently, the aim of the present study is to determine the distribution of RAPTA-T in MDA-MB-231 and MCF-7 cells and to probe whether any difference in distribution exists between these cells possessing different metastatic phenotypes. Both MCF-7 and MDA-MB-231 are breast cancer adenocarcinomas isolated from pleural effusions [18]. MDA-MB-231 are a triple-negative cell line lacking oestrogen and progesterone receptors in which the human epidermal growth factor receptor 2 (HER2/Neu) is not amplified, making it resistant to most chemotherapeutic agents. These characteristics differ to MCF-7 cells, which are positive for both oestrogen and progesterone receptors, and are therefore sensitive to hormonal-based therapies [19].

## 2. Results

**Figure 1:**
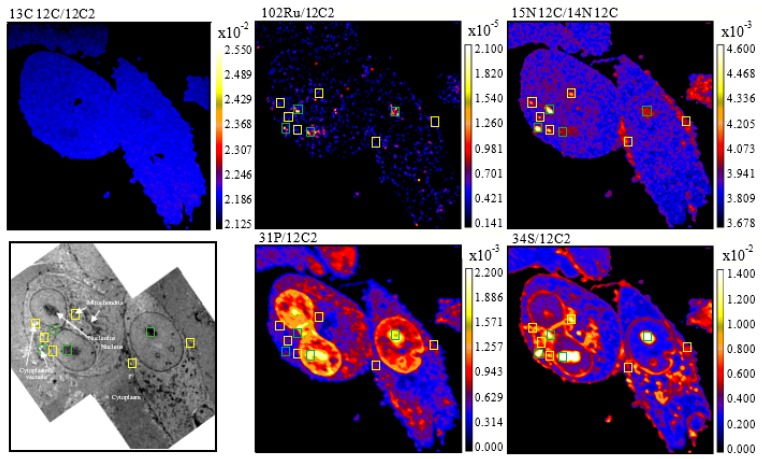
NanoSIMS secondary ion maps of ^31^P^−^/^12^C_2_^−^, ^34^S^−^/^12^C_2_^−^, ^14^N^12^C^−^/^12^C_2_^−^, ^15^N^12^C^−^/^14^N^12^C^−^, ^102^Ru^−^/^12^C_2_^−^, and ^13^C^12^C^−^/^12^C_2_^−^ and TEM images of MDA-MB-231 cells treated with ^15^N/^13^C-labelled RAPTA-T (500 μM, 24 h). Blue boxes indicate Ru-enriched hotspots, yellow boxes indicate ^15^N-enriched hotspots, and green boxes indicate hotspots co-enriched with ^15^N and Ru. Cellular organelles are labeled in the TEM image.

Secondary ion maps of ^13^C^12^C^−^/^12^C_2_^−^, ^14^N^12^C^−^/^12^C_2_^−^, ^15^N^12^C^−^/^14^N^12^C^−^, ^31^P^−^/^12^C_2_^−^, ^34^S^−^/^12^C_2_^−^, and ^102^Ru^−^/^12^C_2_^−^, as well as transmission electron microscopy (TEM) images of MDA-MB-231 cells treated with ^15^N/^13^C-labelled RAPTA-T (500 μM, 24 h), are shown in Figure 1. As observed previously in A2780CR cells treated with ^15^N+^13^C-labelled RAPTA-T [17], ^13^C enrichment was not observed in RAPTA-T treated MDA-MB-231 and MCF-7 cells indicating that the sample preparation dilutes the ^13^C-isotopic enrichment from the ^13^C-enriched toluene ligand to below the detection limit [20]. In the MDA-MB-231 cells, all Ru hotspots found were co-enriched with ^15^N (Figure 1, green boxes), suggesting that the phosphine (PTA) ligand remains coordinated to the Ru centre. However, there were several ^15^N-enriched hotspots that did exhibit Ru enrichment, most likely due to detachment of PTA from Ru.

RAPTA-T was found to accumulate in the nucleolus of MDA-MB-231 cells (Figure 1). This observation is consistent with other studies in which RAPTA-T has been shown to interact with the histone proteins that package and order DNA into nucleosomes [21]. Accumulation of RAPTA-T was also observed on the cell membrane of MDA-MB-231 cells where it could interact with extracellular cell adhesion proteins implicated in its anti-metastatic activity [11]. Overlaying the ^102^Ru^−^ and ^12^C^15^N^−^ maps with TEM images reveals that RAPTA-T also accumulate partially in cytoplasmic vacuoles, which are potential drug targets [22,23], and in mitochondria. The distribution and action of RAPTA-T in mitochondria has been reported previously, where treatment with the drug resulted in an appreciable accumulation in mitochondrial fractions from A2780CR cells [24] and results in perturbation of the expression of several mitochondrial proteins [25]. RAPTA-T accumulation tends to correlate with the sulphur-rich regions of the MDA-MB-231 cells, which is not surprising considering that most organelles in which RAPTA-T is distributed contain sulphur-rich biomolecules.

**Figure 2:**
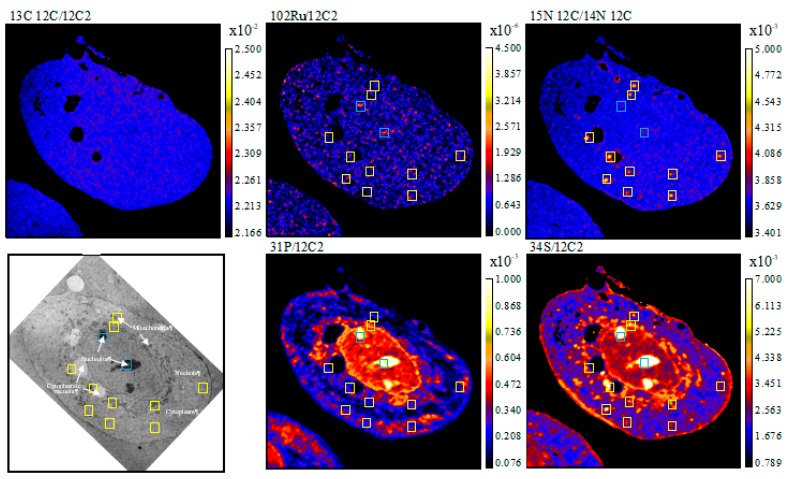
NanoSIMS secondary ion maps of ^31^P^−^/^12^C_2_^−^, ^34^S^−^/^12^C_2_^−^, ^14^N^12^C^−^/^12^C_2_^−^, ^15^N^12^C^−^/^14^N^12^C^−^, ^102^Ru^−^/^12^C_2_^−^, and ^13^C^12^C^−^/^12^C_2_^−^ and TEM of MCF-7 cells treated with ^15^N and ^13^C-labelled RAPTA-T (500 μM, 24 h). Blue boxes indicate Ru enriched hotspots, yellow boxes indicate ^15^N enriched hotspots and green boxes indicate hotspots co-enriched with ^15^N and Ru. Cellular organelles are labeled in the TEM image.

In MCF-7 cells, the accumulation profile of RAPTA-T is in part similar to that in MDA-MB-231 cells, i.e., with accumulation in the nucleolus and a general co-accumulation of the drug at sulphur-rich hotspots (Figure 2). However, in contrast to MDA-MB-231 cells, accumulation of RAPTA-T was not observed in the nucleus or on the cell membrane of MCF-7 cells. From the overlaid TEM images, RAPTA-T was also found to accumulate partially in mitochondria and cytoplasmic vacuoles. The lack of distribution in the nucleus and membrane of MCF-7 cells could partially explain the weaker activity of RAPTA-T in preventing migration, detachment, and reattachment of these cells compared to MDA-MB-231 cells.

## 3. Discussion

Accumulation of RAPTA-T in the membrane of human breast cancer cell lines is significantly higher in the invasive MDA-MB-231 cell line compared to MCF-7 cells. Such differences in RAPTA-T accumulation must be due to differences in the cell type and phenotype. It has been shown previously that A2780CR cells, unlike their cisplatin-sensitive (A2780) counterparts, undergo metastasis and shorten survival rates of mice xenografted with these cells [26]. Hence, both A2780CR and MDA-MB-231 cells are highly invasive, and the selective membrane association of RAPTA-T with these cell lines might be correlated with the anti-metastatic properties of the compound. This selectivity is exemplified by the lack of membrane accumulation of RAPTA-T on the less invasive MCF-7 cells. Notably, in the A2780CR and MDA-MB-231 cell lines, the amount of RAPTA-T associated with the membrane exceeds that inside the cells.

The distribution of RAPTA-T inside both cell lines is largely associated with accumulation in the nucleolus. Interestingly, RAPTA-C, a closely related compound to RAPTA-T, has been shown to reduce proliferation, migration, and tube formation in endothelial cells and also stimulate apoptosis [27]. These effects may be attributed to interactions of RAPTA-C with the endothelial cell membrane and to epigenetic factors.

Overall, the differences observed in the NanoSIMS studies provide new insights into how RAPTA-T distribution correlates with the phenotypic changes induced by its activity on cancer cells. These data emphasise the role of targeting molecules to the cell membrane for the control of metastasis of solid tumours. This aspect has already been stressed for the ruthenium(III) drug, NAMI-A, another potent anti-metastatic drug, which has been shown to bind to integrins [28]. If it is found that RAPTA-T is also able to target integrins, integrin modulation could become a highly attractive approach for tumour control with metal-based drugs. Such a mechanism, which is profoundly different to the development of DNA-damaging metal-based drugs [29], would stimulate the search for novel, selective drugs to control tumour malignancy.

## 4. Materials and Methods

### 4.1. Synthesis and Characterisation of ^13^C/^15^N Labelled RAPTA-T

**Scheme 2:**
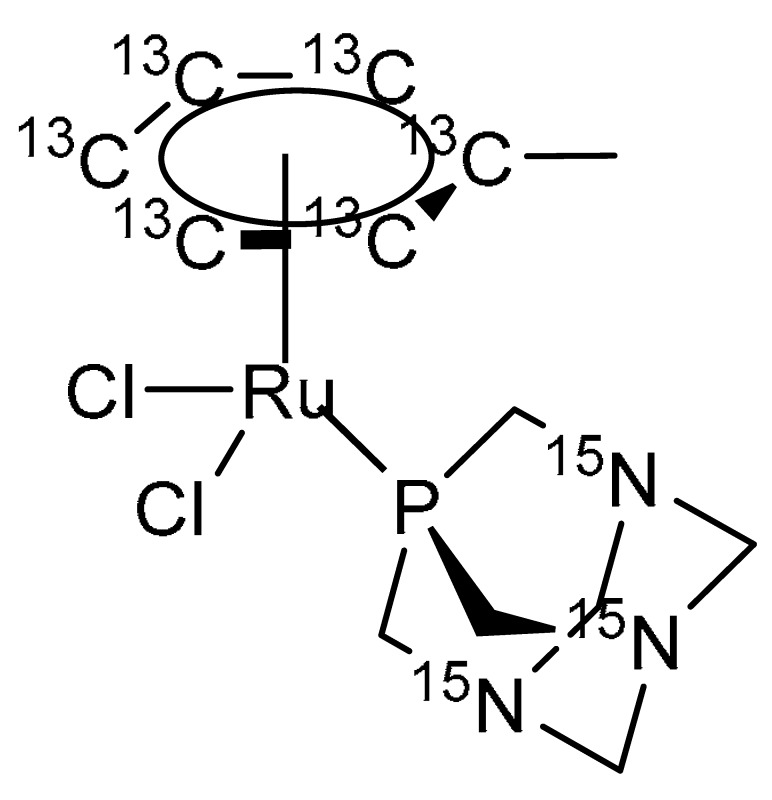
Structure of ^15^N/^13^C labelled RAPTA-T. Characterisation: **^1^**H NMR (400 MHz, Methanol-*d*_4_) = δ 5.95–5.22 (m, 5H), 4.60 (s, 6H), 4.35 (s, 6H), 2.17 (s, 3H). ^13^C NMR (101 MHz, Methanol-*d*_4_) only enriched ^13^C = δ 108.33, 88.64–85.31 (m), 77.87–75.73 (m). ^31^P NMR (162 MHz, Methanol-*d*_4_) = δ 33.43. HRMS (ESI+) *m*/*z* calculated for C_7_^13^C_6_H_20_Cl^15^N_3_PRu [M-Cl+H]^+^: 395.0239; found: 395.0242.

^15^N enriched 1,3,5,7-tetraazatricyclo[3.3.1.1 (3,7)]decane (PTA) was synthesized according to a literature method [30], with minor modifications consisting in the replacement of ^14^NH_4_OH with ^15^NH_4_OH in the described procedure [31]. ^13^C labelled metyl-cyclohexadiene was prepared from a birch reduction of toluene-(phenyl-^13^C6) and used to prepare ^15^N/^13^C-RAPTA-T (Scheme 2) as described previously [17].

### 4.2. Cell Culture

MDA-MB-231 and MCF-7 (human breast adenocarcinoma) cells were cultured in DMEM medium supplemented with 10% foetal calf serum, penicillin 100 units/mL, and streptomycin 100 μg/mL (Invitrogen, Carlsbad, CA, USA). Cells were incubated at 37 °C in a humid environment containing 5% CO_2_.

### 4.3. Cell Preparation

Cells were seeded 50,000 cells/well in 24-well or 500,000 cells/well in 6-well clear bottom plates fitted with sapphire disks. After 24 h, cell media was aspirated and fresh media containing ^15^N and ^13^C-RAPTA-T (500 μM) was added (a high concentration of compound was used due to the reduced incubation time). Upon incubation, the sapphire disks were removed from the media and then high pressure frozen (Leica HPM100, Leica Microsystems, Wetzlar, Germany) with excess 20% BSA solution in 0.01 M PBS (phosphate buffer solution) to avoid any air bubbles becoming trapped and the formation of ice crystals. The frozen cells were then embedded in resin at low temperature [32]. The sapphire discs were placed on a frozen solution of 1% osmium, 0.5% uranyl acetate, 5% water in acetone. The samples where then warmed to room temperature in an ice bucket containing solid carbon dioxide blocks that were allowed to sublime over a period of 2 h until they reached room temperature. At this point the solution was removed and replaced with dry acetone. After washing twice with acetone, the samples were embedded in increasing concentrations of epon resin in acetone. At 100% concentration of resin, the samples were then left overnight to fully infiltrate and then polymerised in a 60 °C oven for at least 12 h. Samples where then glued to empty resin blocks, trimmed, and sections of alternating thickness of 500 nm and 50 nm cut sequentially from the face. The thicker sections were collected onto a glass coverslip stained with 1% touldine blue and imaged with light microscopy and NanoSIMS. The 50 nm thick sections were collected on to an electron microscopy slot grid ready for imaging with transmission electron microscopy at a final magnification of around 1400 times (Tecnai Spirit, FEI Company, Eindhoven, The Netherlands).

### 4.4. NanoSIMS Analysis

NanoSIMS measurements were performed at the Laboratory of Biological Geochemistry, EPFL and the University of Lausanne. Prior to NanoSIMS imaging the samples were gold-coated in order to avoid charging effects. Before acquiring an image, Cs^+^ ions were implanted into the surface of the sample in order to enhance the ionization of the element of interest. The electron multiplier detectors were set up to measure ^12^C_2_^−^, ^13^C^12^C^−^, ^12^C^14^N^−^, ^12^C^15^N^−^, ^31^P^−^, ^34^S^−^, and ^102^Ru^−^ secondary ions, generated by bombarding the sample with a ~4 pA Cs^+^ primary beam focused to a spot size of approximately 160 nm. In order to resolve possible isobaric interferences, the instrument was operated at a mass-resolving power (MRP) of about 10,000. Due to the low signal of ^102^Ru^−^ obtained from cells, peak-shape and mass resolving power was checked using a Ru standard. Data acquisition was performed by scanning the Cs^+^ primary beam over areas of 34 × 34 μm with a 256 × 256 pixel image resolution. The per pixel dwell time of the primary ion beam was 10 ms. The final images are the accumulation of 120 layers obtained by sequential scanning and correspond to a cumulated acquisition time per pixel of 1.2 s. Between every layer, the transmission of the secondary ion beam was optimized and automatic peak centring was performed for ^12^C_2_^−^, ^13^C^12^C^−^, ^12^C^14^N^−^, ^12^C^15^N^−^. The Ru peak could not be centred due to the low count rates. However, post-analysis checks revealed that there was no significant change in the peaks position during the entire acquisition time. The total acquisition time including the centring procedure was 22 h per image.

### 4.5. Data Extraction and Image Processing

NanoSIMS image processing was performed with L’image (L. Nittler, Carnegie Institution of Washington, Washington, DC, USA). Over the ~20 h of image acquisition, the image drift of a 34 × 34 μm image was less than 7 pixels (i.e., less than 1 μm). The data reduction software can easily correct for such a drift by aligning the positions of identified structures. Regions of interest (ROI) were defined manually based on identifiable cell features on the ^31^P^−^ elemental map. Images were accumulated from planes where accumulated counts per ROI were stable with ^12^C^14^N^−^ used as the alignment mass. All other elements were normalized against ^12^C_2_, the images of which are essentially flat, to normalize out small ionization variations across the sample surface.
